# Network Toxicology Integrates In Vivo Evidence Linking Impaired Autophagic Clearance Involving the MTOR-TFEB Axis to Polystyrene Nanoplastic-Induced Neurotoxicity

**DOI:** 10.3390/toxics14090801

**Published:** 2026-09-09

**Authors:** Na Tang, Chun Wang, Meng Zhang, Yajie Li, Yongkang Liang, Jingjing Zhang, Qiang Niu

**Affiliations:** 1 School of Public Health, Shihezi University, Shihezi 832000, China; 2 Key Laboratory for Prevention and Control of Emerging Infectious Diseases and Public Health Security, Shihezi 832000, China

**Keywords:** PS-NPs, neurotoxicity, MTOR-TFEB signaling pathway, impaired autophagic clearance, network toxicology

## Abstract

Long-term exposure to polystyrene nanoplastics (PS-NPs) causes neurotoxicity, but the underlying mechanisms remain unclear. We combined network toxicology, molecular docking, and in vivo experiments to investigate the role of MTOR-TFEB-regulated autophagy in PS-NP-induced neurotoxicity. Potential targets related to PS-NPs and neurodegenerative diseases were screened from public databases. Enrichment analysis indicated involvement of neurodegenerative and autophagy pathways. Protein–protein interaction and docking simulations prioritized *MTOR* as a candidate target. Sprague–Dawley rats were gavaged with PS-NPs (0.15 or 1.5 mg/kg) for 60 days. Morris water maze tests showed impaired spatial learning and memory. Western blotting of hippocampal tissues revealed increased p-MTOR/MTOR ratios, decreased total cytoplasmic and nuclear TFEB, reduced lysosomal proteins (LAMP2, CTSD, and CTSB), elevated autophagy markers SQSTM1 and MAP1LC3B-II, and altered apoptosis regulators (BAX up and BCL2 down). Collectively, PS-NPs disrupt the MTOR-TFEB axis, impair lysosomal function and autophagic clearance, and promote apoptosis, leading to neurocognitive deficits. These findings provide mechanistic insights into the MTOR-TFEB axis and highlight it as a candidate pathway warranting further evaluation as a potential intervention target.

## 1. Introduction

Microplastics (MPs), plastic particles less than 5 mm in diameter, originate from the gradual fragmentation of plastic waste driven by mechanical stress, solar radiation, and biological factors [1]. When MPs undergo mechanical wear or photoinitiated oxidative degradation, they break down into smaller particles, known as nanoplastics (NPs), each smaller than 1 μm in diameter [2]. Due to their significant specific surface areas and hydrophobic nature [3], NPs possess a strong capacity to sorb, bioaccumulate, and transport environmental pollutants, thus acting as potentially effective carriers for airborne, drinking-water, and food pollution, which amplifies the health risks associated with NP exposure [4]. Moreover, smaller particle sizes are associated with higher toxicity [2]. In natural environments, NPs are introduced into the human body through various routes, including the marine food web, diet, and inhalation [5]. Studies have detected NPs in coastal areas, open oceans, and marine organisms such as phytoplankton, mussels, and fish. These organisms ultimately enter the human food chain, posing risks to human health [6]. Furthermore, airborne NPs can be directly inhaled, accumulate in the respiratory tract, and cause respiratory diseases; they may also traverse the blood–brain barrier (BBB) and cause neurotoxicity [7]. In recent years, microplastic pollution has been recognized as an emerging environmental issue [8]. Therefore, it is imperative not to overlook the ecological and toxicological effects of NPs.

NPs can traverse the BBB and become accumulated in the brain, leading to neurotoxicity [9]. Studies have shown that NPs activate brain-resident immune cells, such as astrocytes and microglia, disrupt the BBB, and damage neurons, ultimately contributing to neurodegeneration [10]. These mechanisms are thought to include oxidative stress, neuroinflammation, neuronal apoptosis, autophagy, and mitochondrial dysfunction [11]. In the brains of individuals with dementia, NPs accumulate to a greater degree, with notable deposition in blood vessel walls and within immune cells [12]. A case-control study of 28 cerebrospinal fluid samples from 14 patients with central nervous system (CNS) infections and 14 controls revealed that only polyethylene, polypropylene, polystyrene, and polyvinyl chloride were able to selectively penetrate the human CNS [13]. Animal experiments have demonstrated that polystyrene nanoplastics (PS-NPs) induce neurotoxicity and abnormal brain development in mice [7] and accumulates in the brains of mice, leading to a significant decline in cognitive ability. Furthermore, compared to males, PS-NPs have a more severe impact on the female zebrafish, disrupting the balance of female hormones and interfering with the reproductive process of the zebrafish [14]. In vitro studies further revealed that PS-NPs accumulate in SH-SY5Y cells, including in mitochondria, leading to reduced cell viability and cytotoxicity [15]. Although accumulating evidence indicates that NPs may exert neurotoxic effects, the exact mechanisms through which NPs induce neurotoxicity are yet to be elucidated.

The regenerative capacity of nerve cells is extremely limited, and their normal functions largely depend on basal autophagy [11]. Autophagy-mediated clearance of harmful substances is therefore essential for maintaining neuronal homeostasis. Autophagy is a process of intracellular component degradation, in which “autophagosomes” form to transport damaged materials—including cytoplasmic contents, organelles, membrane fragments, proteins, and nucleic acids—to the lumen of the lysosome for breakdown and recirculation. This mechanism plays a vital role in cellular self-repair and the elimination of dead cells [16]. Autophagic flux refers to the dynamic process of autophagy, which encompasses the generation and maturation of autophagosomes, their fusion with lysosomes, and the subsequent degradation of autophagic substrates within lysosomes [17]. Blockage of autophagic flux can lead to the accumulation of pathogenic proteins and organelle damage, disrupting intracellular homeostasis and causing neuronal injury, thereby contributing to various neurodegenerative diseases [11,18]. The mechanistic target of rapamycin (MTOR) is a highly conserved serine/threonine protein kinase that governs cell growth, cell cycling progression, nutritional intake, protein synthesis, and autophagy [19]. Studies have suggested that MTOR may be the central molecule controlling the initiation and termination of autophagy. MTOR negatively regulates autophagy during the initiation and formation phases, as well as during the processes of autophagosome degradation and lysosomal dynamics [20]. MTOR suppresses autophagy via two principal routes: it directly represses Unc-51-like kinase 1 (ULK1), an essential initiator of autophagy, and it obstructs the nuclear import of transcription factor EB (TFEB), consequently compromising lysosome biogenesis and indirectly attenuating autophagy [21]. TFEB serves as a master governor of autophagic and lysosomal biogenesis and plays a pivotal role in the clearance of protein aggregates [22]. It has been demonstrated that maltitol facilitates TFEB nuclear translocation through activation of the AMPK/MTOR pathway, enhances lysosomal function, triggers autophagy, augments autophagic flux, and thereby attenuates PS-NP-induced apoptosis in intestinal cells [23]. The key nodal alterations induced by polystyrene nanoplastics (PS-NPs) in this signaling pathway within the hippocampal tissue remain to be systematically verified in vivo. In the present study, we comprehensively evaluated this pathway by integrating network toxicology prediction and in vivo animal experiments.

In this study, we selected PS-NPs as a representative plastic polymer because they are used in packaging products such as caps and bottles and represent one of the most common and widely distributed NPs worldwide [4,24]. Our aim was to employ a network toxicology approach to identify the enrichment of potential targets related to PS-NP-induced neurotoxicity within the MTOR-TFEB-regulated autophagy pathway and to pinpoint key genes. Additionally, molecular docking simulations were performed to examine the binding interactions between PS-NPs and the characterized key genes. Furthermore, we established an animal model of PS-NP exposure to validate the aforementioned findings. It is expected that our findings will offer novel mechanistic perspectives on the pathogenesis of PS-NP-induced neurotoxicity and will suggest candidate mechanistic pathways for further investigation.

## 2. Materials and Methods

This research explores the neurotoxic mechanism of PS-NPs by combining network toxicology prediction, molecular docking simulations and in vivo animal assays. The overall study design and technical route are briefly described below. For database search parameters, software operation procedures, detailed experimental protocols, material manufacturers and formulations, and analysis details, please refer to the Appendix A.

### 2.1. Characterization of PS-NPs

Scanning electron microscopy (SEM) and transmission electron microscopy (TEM) were employed to characterize the morphology and surface features of PS-NPs. The PS-NP suspension was prepared by ultrasonication prior to administration, and its colloidal stability was evaluated by zeta potential measurement.

### 2.2. Toxicity Prediction and Analysis of PS-NPs

The molecular structure of PS-NPs was obtained from the PubChem database. ADMETlab 3.0 and ProTox 3.0 platforms were employed to conduct preliminary prediction of its toxicity characteristics.

### 2.3. Target Screening of PS-NPs

Multiple databases, including ChEMBL, SwissTargetPrediction, and STITCH, were used to screen potential human protein targets of PS-NPs. All collected targets were standardized and deduplicated using the UniProt database. Since the ChEMBL, SwissTargetPrediction and STITCH databases are primarily designed for small-molecule compounds, styrene monomer was used as the structural input of PS-NPs, given that the chemical moieties on the surface of polystyrene nanoplastics are identical to those of styrene monomers. Comparable strategies have been adopted in recent toxicological investigations of nanoplastics [25,26]. Furthermore, genes associated with polystyrene were retrieved from the CTD database and integrated with the aforementioned prediction results. All candidate targets were standardized and deduplicated using the UniProt database.

### 2.4. Collection of Neurotoxicity-Related Targets

Neurotoxicity-related genes were retrieved from the CTD, OMIM and GeneCards databases based on specified keywords. Candidate targets mediating PS-NP-induced neurotoxicity were obtained through cross-analysis.

### 2.5. Functional and Pathway Enrichment Analysis

GO functional annotation and KEGG pathway enrichment analysis were performed on candidate targets using Metscape, and a *q*-value < 0.05 was defined as the threshold for statistical significance.

### 2.6. Gene Interaction Network Construction and Core Target Screening

Under the high-confidence interaction threshold (0.700), a PPI network was constructed in the STRING database. The Cytoscape software (version 3.9.1) was used to screen core hub targets via topological analysis.

### 2.7. Molecular Docking Verification

Given that conventional molecular docking software has not been developed to characterize interactions between nanoparticles and proteins, a styrene 20-mer was adopted in this study to mimic the surface chemical structure of PS-NPs for molecular docking simulations [27]. The kinase domain of *MTOR* (PDB ID: 4JSV) and full-length *TFEB* (PDB ID: 7Y62) were selected as target receptors, as these two proteins represent the core molecular targets of interest in the present work. All docking calculations were performed using AutoDock Vina (version 1.1.2).

### 2.8. Experimental Reagents and Antibodies

All main experimental reagents, antibodies and PS-NP samples used in this study were purchased from corresponding commercial manufacturers.

### 2.9. Animal Experiments

Adult Sprague–Dawley rats (180–220 g, 1:1 male-to-female ratio) free of specific pathogens were purchased from Beijing Saibainuo Biotechnology Co., Ltd. (License No.: SYXK (Beijing, China) 2024-0001). Animals were housed in plastic cages under controlled environmental conditions (12/12 h light–dark cycle; humidity, 50–60%; temperature, 20–25 °C), with ad libitum access to standard chow and double-distilled water, as well as environmental enrichment materials (e.g., bedding and plastic tunnels). After one week of acclimation, rats were randomly divided into three groups (*n* = 12 per group): a control group (0 mg/kg), as well as a low-dose group (0.15 mg/kg) and a high-dose group (1.5 mg/kg) exposed to PS-NPs (particle size, 80 nm). PS-NPs were administered orally once daily for 60 consecutive days. Doses were selected based on environmentally relevant human exposure levels and converted across species using body surface area normalization; detailed methods are provided in the Supplementary Materials. Body weight and general health status were monitored weekly. Preset humane endpoints included severe weight loss (>20% of initial body weight), persistent lethargy, or inability to consume food or water. None of the animals reached these endpoints. Throughout the experiment, no deaths or obvious signs of stress (such as piloerection, a hunched posture, or reduced activity) were observed. For the Morris water maze test, six rats per group (equal numbers of males and females) were randomly selected. Following behavioral testing, all rats were deeply anesthetized by intraperitoneal injection of sodium pentobarbital (60 mg/kg) and euthanized by decapitation. Hippocampal tissues were rapidly dissected on ice, immediately frozen in liquid nitrogen, and stored at −80 °C. All animal procedures were approved by the Biological Ethics Committee of Shihezi University (Approval No.: A2024-746; approved in February 2024) and conducted strictly in accordance with the ARRIVE guidelines.

### 2.10. MWM Test

The MWM experiment consists of the Position Navigation Test (PNT) and the Spatial Exploration Test (SPT). The PNT is used to assess the spatial learning and memory abilities of rats, while the SPT is used to evaluate memory storage and retrieval capabilities. Relevant behavioral indicators were recorded and analyzed.

### 2.11. Western Blot Detection

For Western blot analysis, each group consisted of 3 independent rats, resulting in 3 sets of replicate results. Quantitative data are presented as the mean ± SD. Western blot was performed to detect the expression levels of the MTOR-TFEB pathway, lysosomal function, autophagy and apoptosis-related proteins in the hippocampal tissues of rats. A professional image processing software was used for protein quantification and band gray analysis.

### 2.12. Statistical Analysis

All data calculations were performed using the SPSS 26.0 software. Repeated measures analysis of variance and one-way analysis of variance were used for data comparison. Data are presented as the mean ± SD, and *p* < 0.05 was considered as the threshold for statistically significant differences. Due to the limited sample size in each gender subgroup in the water maze test (*n* = 3 per gender per group), sex was not included as an independent factor in the primary statistical model. Data from all male and female rats were combined for final analysis, as this study was underpowered to detect a treatment × gender interaction.

## 3. Results

### 3.1. Characterization of PS-NPs, Toxicity Prediction and Collection of Targets

**Figure 1:**
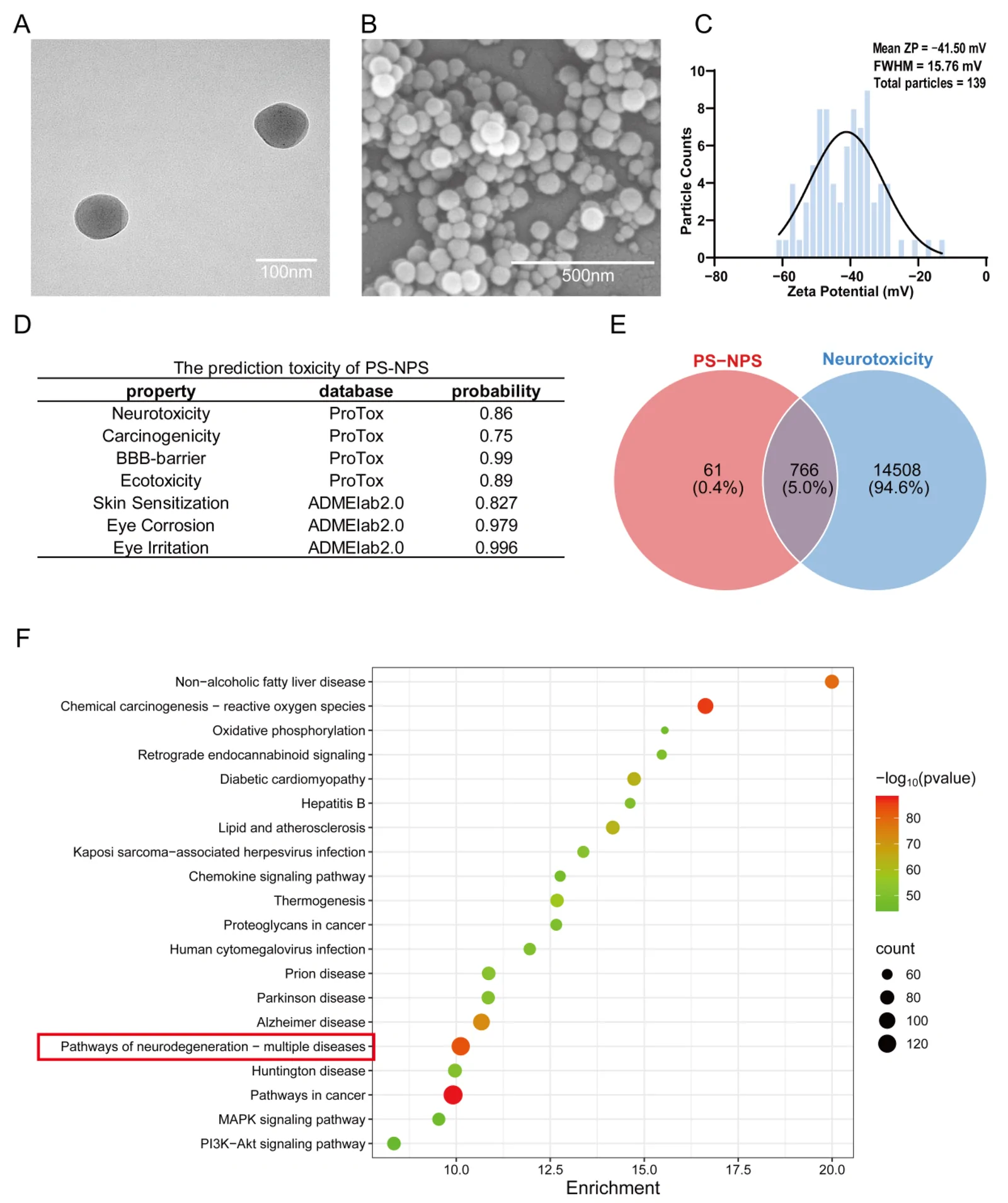
Characterization of PS-NPs, toxicity prediction and collection of targets. (**A**) Scanning electron microscopy (SEM) image showing the spherical morphology and surface features of PS-NPs. Scale bar = 100 nm. (**B**) Transmission electron microscopy (TEM) image revealing the ultrastructure and dispersion state of PS-NPs. Scale bar = 500 nm. (**C**) Zeta potential distribution profile of PS-NPs in aqueous solution. The mean zeta potential was −41.50 mV, with a full width at half maximum (FWHM) of 15.76 mV (total particle counts = 139). The negative zeta potential indicates a negatively charged surface, suggesting good dispersion stability. (**D**) Toxicity prediction results of PS-NPs. (**E**) Venn diagram of toxicity targets (PS-NPs) and disease targets (neurotoxicity) after merging and removing duplicates (**F**) Kyoto Encyclopedia of Genes and Genomes (KEGG) pathway enrichment analysis. The “Pathways of neurodegenerative diseases—multiple diseases” marked in the red box refers to the neurotoxicity-related pathways.

As shown in Figure 1A, TEM micrographs revealed that the PS-NPs were predominantly spherical in shape, with smooth surfaces and well-defined edges. Importantly, the particles were evenly dispersed without significant aggregation. The SEM micrographs revealed that the PS-NPs were predominantly spherical in shape (Figure 1B), with a smooth surface and a mean diameter of approximately 80 nm, consistent with the specifications provided by the manufacturer. The particles were observed to be moderately aggregated, which is likely attributable to the high surface energy of the nanomaterials during the drying process. The surface charge and colloidal stability of the 80 nm PS-NPs were assessed by zeta potential analysis. As shown in Figure 1C, the PS-NPs exhibited a zeta potential of −41.50 ± 1.02 mV in deionized water, indicating a strong negative surface charge and favorable electrostatic repulsion, which ensures good dispersion stability during the subsequent in vivo administration. The toxicity prediction results of PS-NPs are shown in Figure 1D. The results indicate that PS-NPs exhibit neurotoxicity, carcinogenicity, BBB permeability, ecotoxicity, skin sensitization, and ocular corrosive irritation. Among these, the probabilities of ocular damage (corrosion/stimulation) and BBB permeability are close to 1. A combined 863 PS-related targets were identified from the ChEMBL, SwissTargetPrediction, STITCH, and CTD databases. After removing duplicates, 827 targets remained and were designated as “PS-NPs” (Supplementary Document S1). Using the keywords “neurotoxicity syndromes”, “nerve degeneration”, “neuronal apoptosis”, and “cognitive disorder”, we collected and deduplicated targets from the CTD, OMIM, and GeneCards databases, yielding 234, 18,765, 17,139, and 21,173 target genes, respectively. After merging, removing duplicates, and taking the intersection, a total of 15,274 target genes related to neurodegenerative diseases were obtained and named “neurotoxicity” (Supplementary Document S2). The toxic targets (PS-NPs) and disease targets (neurotoxicity) were then merged, had duplicates removed, and screened based on the median, resulting in 766 potential targets that may be associated with PS-NP-induced neurodegenerative disease (Figure 1E).

GO analysis produced 243 cellular components (CCs), 2963 biological processes (BPs), and 412 molecular functions (MFs). KEGG pathway analysis identified 241 significantly enriched pathways. Supplementary Figure S1 shows the enrichment of each gene relative to the top 10 categories (for CCs, BPs, and MFs). Figure 1F presents the 20 most significantly enriched KEGG pathways. Supplementary Figure S2 lists the 241 KEGG pathways analyzed, categorized by Genetic Information Processing, Metabolism, Environmental Information Processing, Organismal Systems, and Human Diseases Cellular Processes [28].

For GO enrichment, BP terms were mainly involved in mitochondrial ATP synthesis coupled with electron transfer and oxidative phosphorylation-related processes. CC terms were enriched in the NADH dehydrogenase complex, respiratory chain complex I, mitochondrial membrane, and various transmembrane transporter protein complexes. MF terms were related to NADH dehydrogenase activity, kinase activity, and various transmembrane transporter protein activities.

The top ten KEGG pathways included: pathways related to cancer; neurodegenerative disease pathways (various diseases); Alzheimer’s disease; Creutzfeldt–Jakob disease; thermogenesis; Kaposi’s sarcoma-associated herpesvirus infection; diabetic cardiomyopathy; chemical carcinogenesis—the reactive oxygen species pathway; and non-alcoholic fatty liver disease. Among these, the “neurodegenerative diseases—various diseases” pathway had a total of 123 enriched genes, and the “autophagy—animal” pathway had 32 enriched genes.

### 3.2. Building the PPI Network and Selecting Core Genes

**Figure 2:**
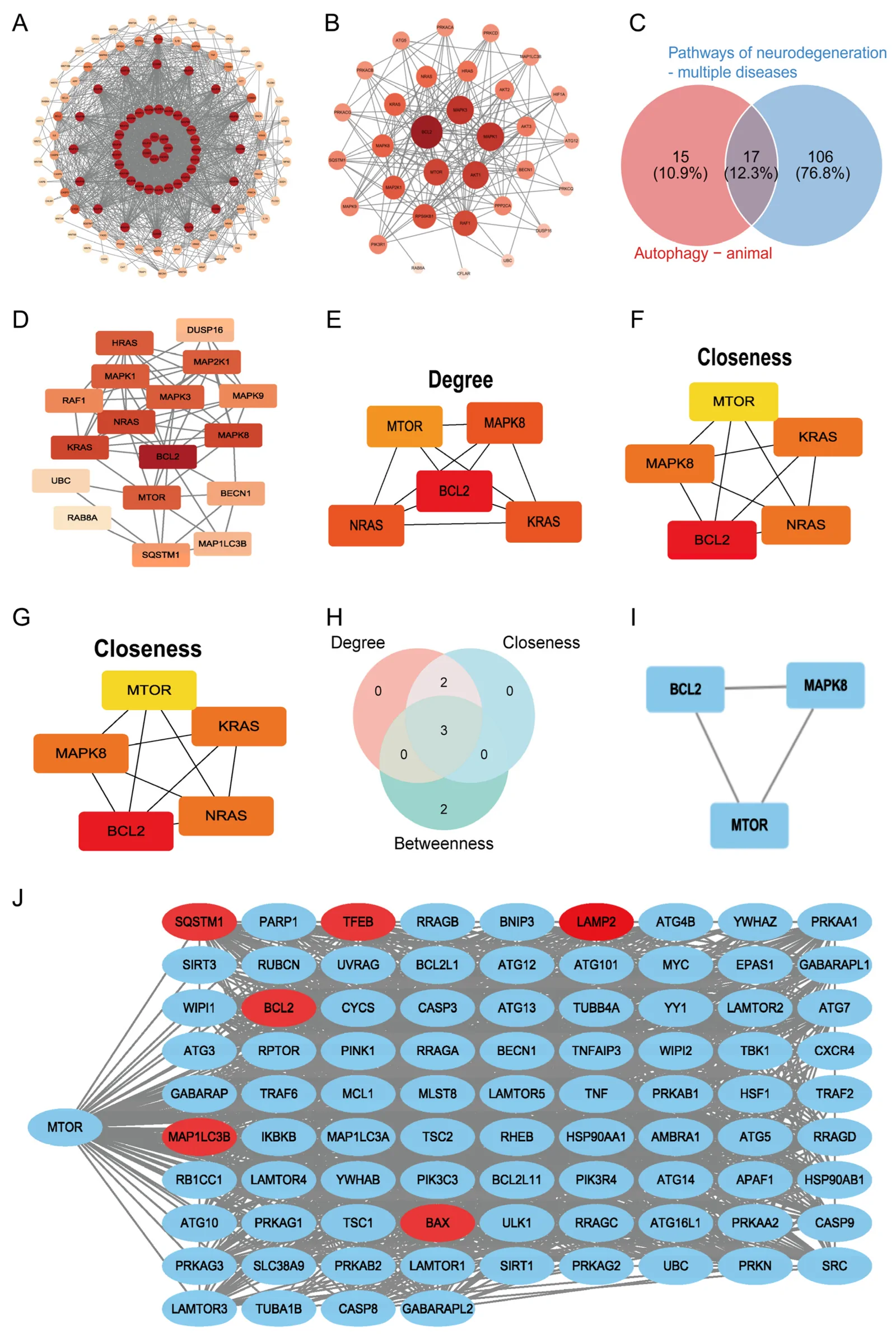
Building the PPI network and selecting core genes. (**A**) PPI network of the neurodegeneration–multiple diseases pathway. (**B**) PPI network of the autophagy–animal pathway. (**C**) Venn diagram of overlapping gene targets between the neurodegeneration–multiple diseases pathway and the autophagy–animal pathway. (**D**) The PPI network of 17 intersection genes. (**E**) Degree analysis results of top five genes. (**F**) Closeness centrality analysis results of top five genes. (**G**) Betweenness centrality analysis results of top five genes. (**H**) Venn diagram of hub genes. (**I**) PPI network of hub genes. (**J**) Interacting genes of *MTOR* with a combined score ≥ 0.5 in the Apoptosis and Autophagy pathway. The red genes are the ones under study in this research.

The STRING database was imported with 123 gene targets that are more frequently found in neurodegenerative signaling pathways and various disease signaling pathways, as well as 32 gene targets that are common to autophagy signaling pathways. Using the STRING website, the PPI network was constructed and subsequently exported to Cytoscape for visualization (Figure 2A,B). The intersection of these two pathways was taken to obtain 17 common intersection gene targets (Venn diagram in Figure 2C). The STRING database was adopted to build the PPI network of relevant targets, which was then imported into Cytoscape for visual analysis (Figure 2D). The betweenness centrality, closeness centrality and degree (the number of connections) algorithms of the CytoHubba plugin were used to screen out the top five core genes (Figure 2E–G). Based on the three algorithms, the three core genes of the intersection were *BCL2*, *MAPK8*, and *MTOR* (Figure 2H,I). On the PathCards website (https://pathcards.genecards.org/; accessed on 3 October 2025), 187 genes of the Apoptosis and Autophagy pathway were retrieved. These 187 genes and *MTOR* were imported into the STRING website for interaction analysis, and the results were exported as Supplementary Data. Genes with a combined score ≥ 0.5 were selected and imported into Cytoscape for visualization (Figure 2J). According to the purpose of this research, *MTOR*, *TFEB*, *LAMP2*, *SQSTM1*, *MAP1LC3B*, *BAX*, and *BCL2* were selected for further study, and *TFEB* is a core transcription factor regulating the autophagolysosomal function downstream of the *MTOR* pathway and is closely related to neurodegenerative changes.

### 3.3. Exposure to PS-NPs Impairs Learning, Memory, and Memory Retention in Rats

**Figure 3:**
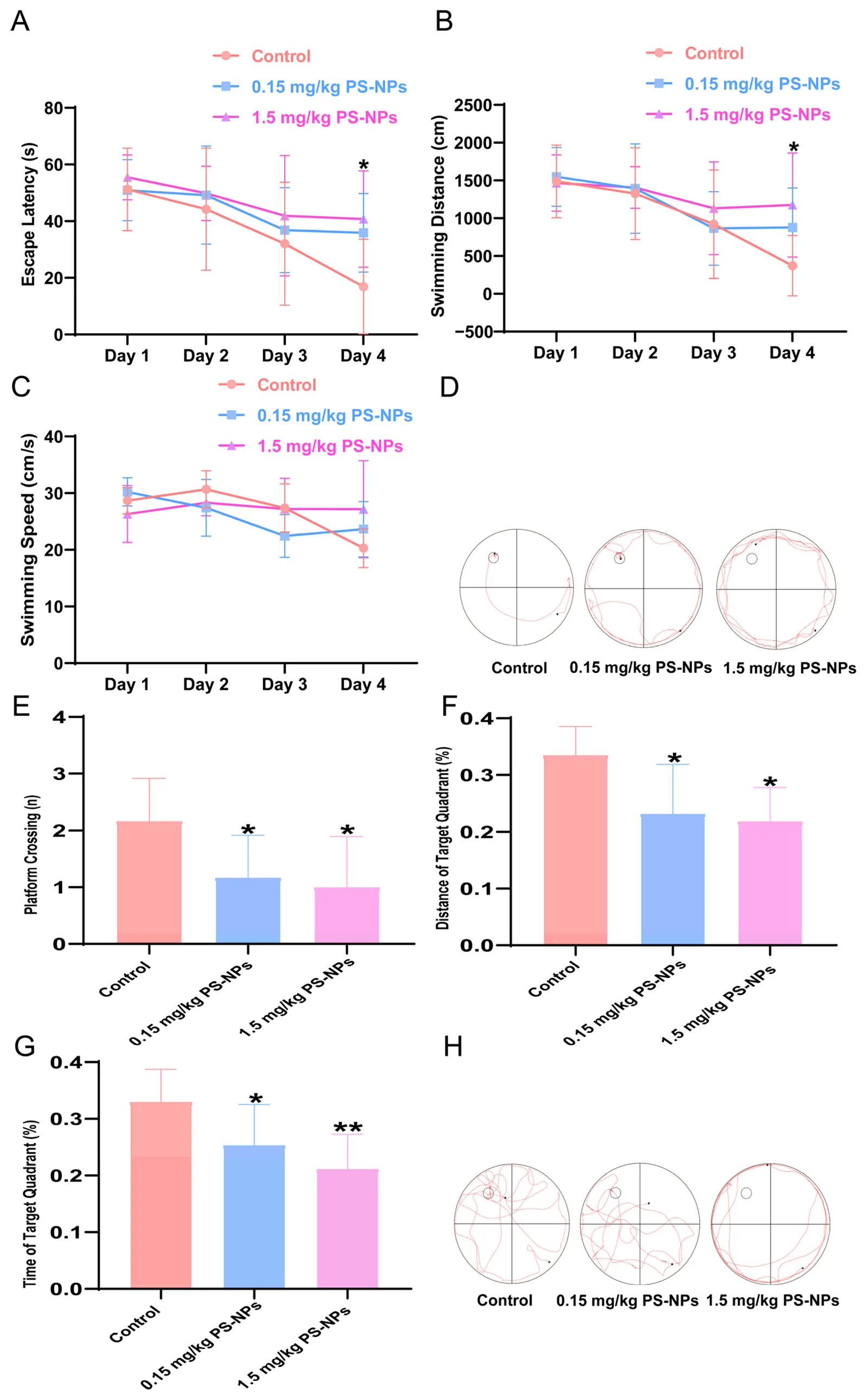
Exposure to PS-NPs impairs learning, memory, and memory retention in rats. (**A**) The mean escape latency to the platform. (**B**) The mean swimming distance to the platform. (**C**) The mean swimming speed to the platform. (**D**) Representative traces in the PNT. The red lines represent the movement paths of the rats, the circles represent the platforms suspended on the water maze, and the dots indicate the rats. (**E**) The number of platform crossings. (**F**) Distance spent in the target quadrant. (**G**) Time spent in the target quadrant. (**H**) Representative traces in the SPT. The red lines represent the movement paths of the rats, the circles represent the platforms suspended on the water maze, and the dots indicate the rats. The data are presented for six rats in each group. * *p* < 0.05 versus the control group. ** *p* < 0.01 versus the control group.

In the PNT, compared with the control group, rats in the groups exposed to 0.15 mg/kg and 1.5 mg/kg of PS-NPs showed significantly prolonged escape latency on the fourth day (*p* < 0.05; Figure 3A), a significantly increased swimming distance (*p* < 0.05; Figure 3B), and no significant difference in swimming speed (*p* > 0.05; Figure 3C). In the control group, the escape latency on the 4th day was shorter compared to the 1st day (*p* < 0.05; Figure S4A), the swimming distance was shorter (*p* < 0.01; Figure S4B), and there was a significant difference in swimming speed between the 4th day and the 1st day (*p* < 0.001; Figure S4C). In contrast, the rats in the 0.15 mg/kg-exposure group showed differences in escape latency, swimming distance, and swimming speed from the 1st day to the 4th day (*p* < 0.05; Figure S4A–C), but the decrease was significantly smaller than that of the control group. While for the group exposed to 1.5 mg/kg of PS-NPs, there was no significant improvement in these indicators from the 1st day to the 4th day (*p* > 0.05; Figure S4A–C). In the SPT, compared with the control group, rats in the groups exposed to 0.15 mg/kg and 1.5 mg/kg of PS-NPs exhibited a significantly reduced number of platform crossings (*p* < 0.05; Figure 3E), and the rats’ dwell time in the target quadrant was significantly reduced, and the proportion of their total movement distance spent there decreased markedly (*p* < 0.05; Figure 3F,G). Representative swimming paths of the PNT and SPT are shown in Figure 3D and Figure 3H, respectively.

### 3.4. PS-NPs Interact with the Key Molecules of the MTOR-TFEB Signaling Pathway

**Figure 4:**
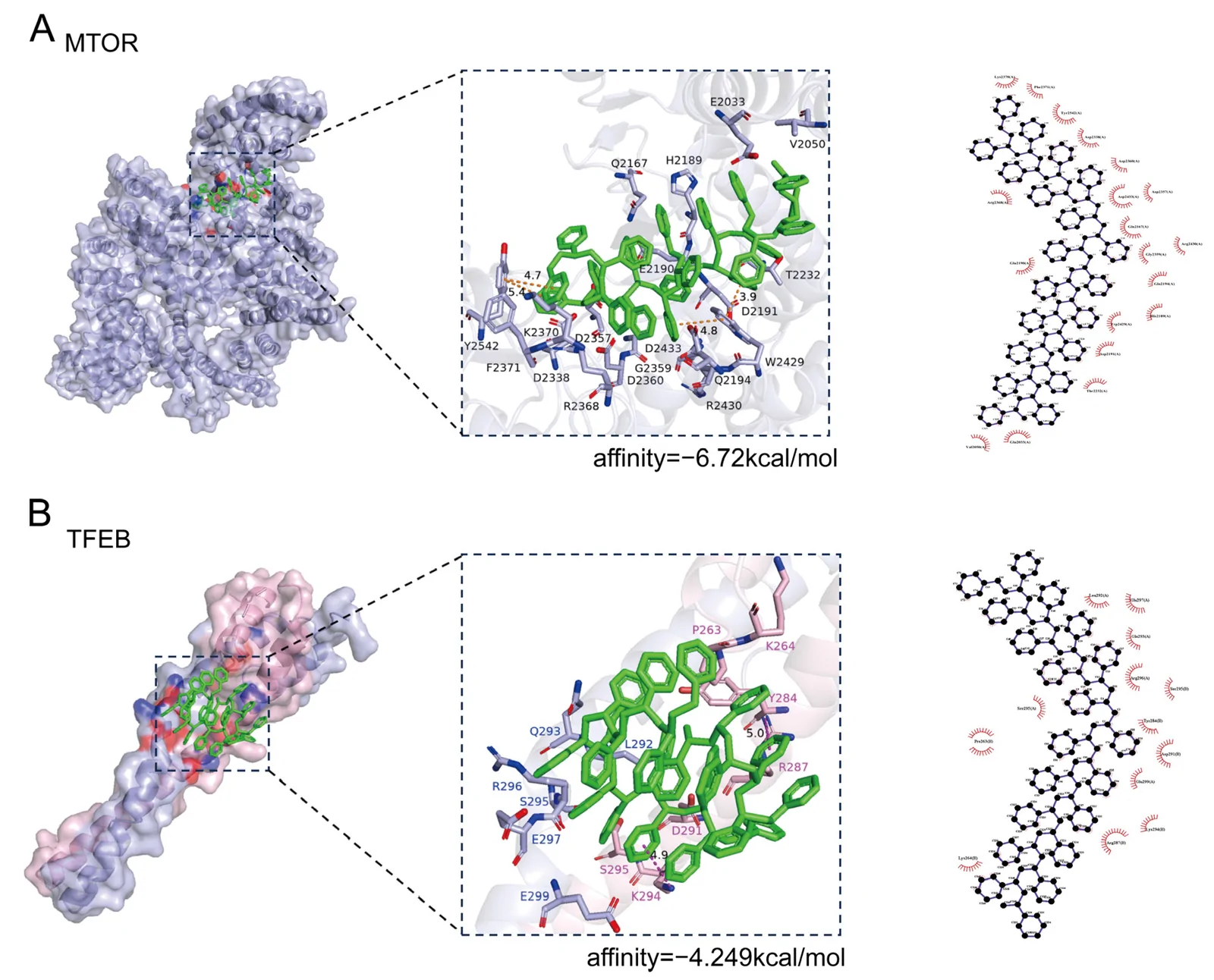
PS-NPs interact with the key molecules of the MTOR-TFEB signaling pathway. (**A**) Docking conformation of the styrene 20-mer with the kinase domain of *MTOR*, showing a binding affinity of −6.72 kcal/mol. (**B**) Docking conformation of the styrene 20-mer with full-length *TFEB*, showing a binding affinity of −4.249 kcal/mol. The green part in the figure represents “styrene 20-mer”.

The interactions between PS-NPs and two key target proteins were evaluated using molecular docking analysis: *MTOR*, *TFEB*. AutoDock Vina was employed to perform molecular docking simulations between the two targets and PS-NPs, and a complete set of docking results was successfully obtained (Figure 4A,B). The binding energy of MTOR is lower than −5.0 kcal/mol, indicating that the styrene oligomer representing the PS–nanoparticles has a good affinity for the *MTOR* target protein in the computational simulations. Notably, the binding energy for *TFEB* (−4.249 kcal/mol) did not reach the predefined −5.0 kcal/mol threshold; *TFEB* was nevertheless retained as a target of interest on the basis of its well-established role downstream of *MTOR* in lysosomal biogenesis, and this docking result should be interpreted as a tentative indication only. Meanwhile, we conducted a docking experiment using styrene with nine target molecules. The results are shown in Supplementary Figure S3. It should be emphasized that these docking results are based on computational predictions using the styrene oligomer as a proxy model for the surface chemical structure of the PS–nanoparticles, and they cannot directly prove the existence of the binding between the nanoparticles and the protein in vivo. The main value of this computational analysis lies in providing a priority ranking basis for subsequent experimental verification of the candidate target.

### 3.5. PS-NPs Affect the MTOR-TFEB Signaling Pathway, Impair Autophagic Clearance, and Lead to Apoptosis

**Figure 5:**
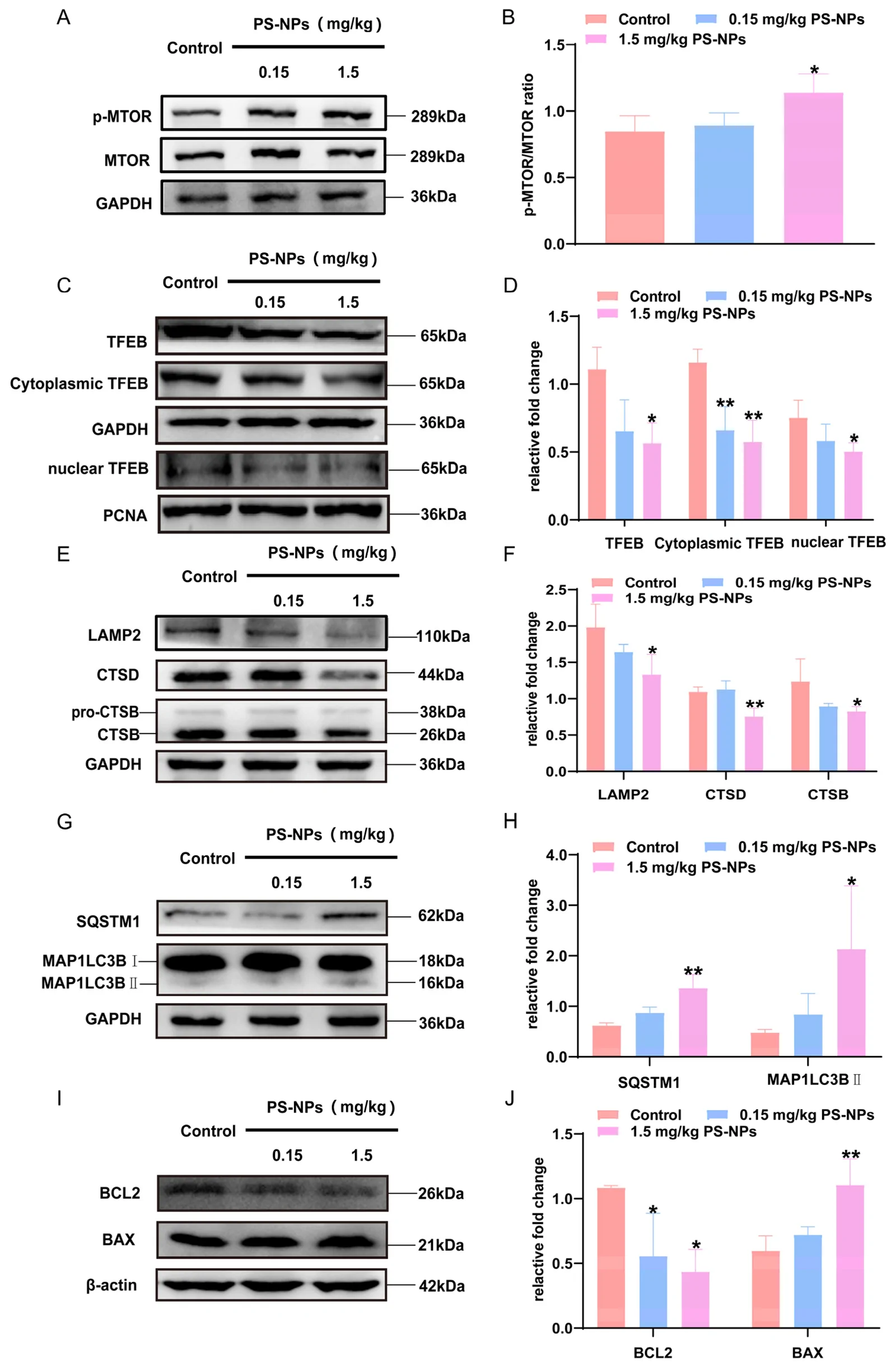
PS-NPs affect the MTOR-TFEB signaling pathway, impair autophagic clearance, and lead to apoptosis. (**A**) Representative Western blot images for MTOR in hippocampal tissues of SD rats. (**B**) Quantitative analyses of MTOR in hippocampal tissues of SD rats. (**C**) Representative Western blot images for TFEB, cytoplasmic TFEB and nuclear TFEB in hippocampal tissues of SD rats. (**D**) Quantitative analyses of TFEB, cytoplasmic TFEB and nuclear TFEB in hippocampal tissues of SD rats. (**E**) Representative Western blot images for lysosomal-associated proteins LAMP2, CTSD and CTSB in hippocampal tissues of SD rats. (**F**) Quantitative analyses of lysosomal-associated proteins LAMP2, CTSD and CTSB in hippocampal tissues of SD rats. (**G**) Representative Western blot images for autophagy markers SQSTM1 and MAP1LC3B in hippocampal tissues of SD rats. (**H**) Quantitative analyses of autophagy markers SQSTM1 and MAP1LC3B in hippocampal tissues of SD rats. (**I**) Representative Western blot images for apoptosis markers BCL2 and BAX in hippocampal tissues of SD rats. (**J**) Quantitative analyses of apoptosis markers BCL2 and BAX in hippocampal tissues of SD rats. The data are presented as means ± SD of three independent biological replicates. * *p* < 0.05 versus the control group. ** *p* < 0.01 versus the control group.

The MTOR-TFEB signaling pathway is a key regulator of autophagic flux [29]. To further investigate the association between PS-NPs and impaired autophagic clearance, we examined the expression of key components of the MTOR-TFEB pathway. Compared with the control group, rats treated with 1.5 mg/kg PS-NPs showed an increased expression level of the p-MTOR/MTOR protein ratio (*p* < 0.05; Figure 5A,B), whereas total TFEB and nuclear TFEB protein levels decreased. Moreover, in rats exposed to 0.15 mg/kg and 1.5 mg/kg of PS-NPs, cytoplasmic TFEB protein levels decreased more significantly (*p* < 0.01 and *p* < 0.05; Figure 5C,D). Notably, when the ratio of nuclear to cytoplasmic TFEB or nuclear to total TFEB was calculated, no significant difference was observed between PS-NPs-treated groups and the control group (*p* > 0.05, Figure S5), indicating that the nuclear translocation machinery per se was not disrupted by PS-NPs. Given that the lysosome serves as the terminal compartment of autophagy, we assessed the levels of lysosome-associated proteins and found that compared with the control group, exposure to 1.5 mg/kg of PS-NPs significantly decreased the protein expression of LAMP2, CTSB, and CTSD (*p* < 0.01 and *p* < 0.05; Figure 5E,F). We next examined autophagy-related markers and found that relative to the control group, the protein levels of SQSTM1 and MAP1LC3B-II were significantly elevated in the hippocampal tissue of PS-NP-treated SD rats (*p* < 0.01 and *p* < 0.05; Figure 5G,H). Finally, an assessment of apoptosis-related markers revealed that compared with the control group, PS-NP-treated rats showed a significant increase in the pro-apoptotic protein BAX and a significant decrease in the anti-apoptotic protein BCL2 in hippocampal tissue (*p* < 0.01 and *p* < 0.05; Figure 5I,J).

## 4. Discussion

NPs have wide applications and can be encountered by people through various routes in daily life; therefore, their toxic effects cannot be ignored. Numerous studies have shown that long-term exposure to NPs interferes with neuronal function and may accelerate brain aging and cognitive decline [27,30]. By integrating network toxicology with in vivo animal experiments, this study demonstrates that PS-NPs are associated with impaired autophagic flux, potentially through interference with the MTOR-TFEB signaling pathway, thereby contributing to neurotoxicity. Core components of this pathway, including MTOR, TFEB, autophagy and lysosomes, are well-established regulators involved in neurodegeneration. The novelty of the present study lies in providing multi-layered in vivo evidence from a hippocampal PS-NP-exposure model, which directly links hub genes predicted via network toxicology to impaired functional autophagic flux and behavioral deficits.

Multiple studies have confirmed that PS-NPs exert neurotoxic effects [10,31]. In a rat model of PS-NP exposure, we found that PS-NPs impaired memory, learning, and memory retention, which is consistent with the report by Jin et al. that exposure to PS-MPs of different particle sizes induced learning and memory deficits and neurotoxicity in mice [32]. Notably, these results align with epidemiological evidence. Xu et al. [33] reported that total microplastic concentrations in the blood are higher in patients with Parkinson’s disease than in healthy controls. These findings collectively indicate that overexposure to PS-NPs impairs neural function.

Autophagic flux, the dynamic process of autophagy, is crucial for maintaining neuronal homeostasis. Moreover, MTOR is the central molecule controlling the initiation and termination of autophagy [20]. TFEB is an important regulator of lysosomal and autophagic function, and its expression is negatively regulated by MTOR [34]. Under physiological conditions, MTORC1 directly phosphorylates TFEB, retaining it in the cytoplasm. Conversely, MTORC1 inhibition drives TFEB nuclear translocation and the transcriptional activation of lysosomal and autophagy-related genes [35]. Liang et al. showed that PS-NPs activate MTOR signaling, thereby suppressing TFEB nuclear translocation [36]. Yang et al. showed that early-life exposure to PS-NPs activates MTOR signaling, resulting in autophagy–lysosome dysfunction and disrupted protein homeostasis [37]. In this study, PS-NPs increased the p-MTOR/MTOR ratio, indicating abnormal activation of MTOR. At the same time, they decreased the levels of total, cytoplasmic and nuclear TFEB proteins. However, the ratios of nuclear/cytoplasmic and nuclear/total TFEB showed an upward trend, but there was no significant difference (Figure S5), suggesting that PS-NPs did not impair the nuclear import machinery but rather caused a total depletion of the TFEB protein pool. Suppression of the autophagy–lysosome pathway in the hippocampus of SD rats upon PS-NP exposure is demonstrated by these findings, thereby severely impairing the capacity of hippocampal neurons to clear misfolded proteins and damaged organelles. Furthermore, PS-NPs can lead to autophagy for the clearance of damage, accompanied by lysosomal dysfunction, suggesting that physical disruption of lysosomal integrity may contribute to the collapse of autophagy–lysosome system function [38].

Lysosomes constitute the ultimate component of the autophagic flux. They degrade and recycle autophagosomal contents, thereby maintaining normal cellular functions [16]. Lysosome-associated membrane protein 2 (LAMP2) is a major constituent of the lysosomal membrane that maintains membrane integrity, shields lysosomes from hydrolase-mediated degradation, and plays a pivotal role in lysosomal biogenesis [23]. Cathepsin D (CTSD), an aspartic protease, and cathepsin B (CTSB), a cysteine protease, are lysosomal acidic hydrolases that mediate the degradation of diverse substrates. Their deficiency can lead to disorders in lysosomal biosynthesis and abnormal lysosomal function, thereby contributing to various neurological diseases [39]. Notably, we observed that PS-NP exposure disrupted lysosomal function, manifested by a decreased expression of LAMP2, CTSD, and CTSB. This finding is consistent with the study by Jin et al. [23], who found that PS-NP exposure reduced LAMP2 protein expression levels. Evidence from Lu et al. [40] also demonstrates that PS-NPs impair lysosomal degradation function, as shown by decreased CTSB and CTSD protein levels, thereby causing renal toxicity. Together, these findings indicate that PS-NPs cause disruption of the lysosomal membrane and reduction in lysosomal degradation capacity, which may exacerbate PS-NP-induced neurotoxicity.

Moreover, PS-NP exposure upregulated the level of MAP1LC3B, an autophagosomal membrane protein essential for autophagosome formation [41]. Therefore, our results indicate that PS-NPs induce autophagosome accumulation. Our data also show an increase in SQSTM1 levels. Since SQSTM1 is a selective autophagy receptor that binds MAP1LC3B and facilitates substrate degradation via autophagolysosomes after autophagosome–lysosome fusion [42], an elevated expression of both MAP1LC3B and SQSTM1 typically indicates impaired autophagic degradation. Consequently, our results suggest that PS-NPs impair lysosomal degradation function, prevent autophagosome degradation, and cause impaired autophagic clearance. In this study, PS-NP exposure upregulated MAP1LC3B-II and SQSTM1 levels in the rat hippocampus, suggesting accumulation of autophagosomes and autophagic substrates. However, because MAP1LC3B-II and SQSTM1 were measured only at steady state, we cannot definitively conclude that autophagic flux is completely blocked. The concurrent decrease in lysosomal proteins (LAMP2, CTSB, and CTSD) supports the interpretation that impaired lysosomal degradation capacity likely contributes to the observed accumulation of autophagic substrates. This is consistent with, but does not independently prove, autophagic flux blockade.

Impaired autophagic clearance ultimately leads to programmed cell death [43]. We found that PS-NPs upregulated the pro-apoptotic protein BAX and downregulated the anti-apoptotic protein BCL-2, indicating that PS-NP exposure triggers apoptosis. Consistent with Jin et al. [23], PS-NPs caused autophagic lysosomal dysfunction, upregulated BAX, downregulated BCL-2, and induced apoptosis in colon cancer cells. Therefore, our findings suggest that PS-NP-induced apoptosis contributes to neurotoxicity.

Based on our results and the existing literature, we propose the following sequence: in the early stage of exposure, PS-NPs are engulfed by neurons via endocytosis and traverse the BBB, accumulating in the autophagosome–lysosome system [12]. This accumulation induces lysosomal stress and membrane damage, thereby triggering a cellular stress response. Initially, it activates MTOR signaling and promotes autophagosome formation in an attempt to clear the foreign particles. However, continuous exposure to PS-NPs leads to a general decrease in the total protein level of TFEB. This depletion, combined with persistent MTOR activation, limits the absolute amount of TFEB that can enter the nucleus, preventing it from reaching the transcriptional threshold required to drive the CLEAR network (e.g., CTSD and CTSB). Indeed, we observed a decreased expression of lysosomal proteins (LAMP2, CTSB, and CTSD), confirming insufficient lysosomal biogenesis. Under certain oxidative or lysosomal stress conditions, although the total TFEB decreases, the ratio shows no statistically significant difference, and there is a potential upward trend, which may reflect a compensatory stress response, enhancing nuclear retention or slowing down nuclear export [44]. Nevertheless, the absolute nuclear TFEB concentration falls below the functional threshold, leading to impaired lysosomal function, an accumulation of autophagic substrates (SQSTM1 and MAP1LC3B-II), ultimately activating the mitochondrial apoptotic pathway (BAX up and BCL2 down) and causing neurotoxicity. It must be acknowledged that this study did not directly measure PS-NPs in the hippocampal tissue. Our inference regarding the passage of PS-NPs through the blood–brain barrier and their accumulation in the brain was based on indirect evidence from the literature. In the future, it is still necessary to use radioactive tracing or advanced imaging techniques to directly quantify the PS-NPs in brain tissue in order to clearly confirm their presence in the hippocampus.

## 5. Conclusions

In conclusion, our research has demonstrated through in vivo experiments that PS-NPs can deregulate the MTOR-TFEB signaling axis, leading to lysosome dysfunction via total TFEB depletion rather than nuclear translocation blockade, thereby impairing autophagic clearance, thereby inducing apoptosis of hippocampal neurons and ultimately resulting in cognitive function deficits. These findings establish the MTOR-TFEB pathway as a promising candidate mechanism pathway, providing new experimental clues for an in-depth exploration of the molecular basis of the neurotoxicity of PS-NPs. However, since this study did not conduct targeted intervention or pathway rescue experiments, the feasibility of this pathway as a therapeutic intervention target needs to be further verified in subsequent studies through strategies such as gene knockout and pharmacological inhibition or activation.

## Data Availability

The raw data supporting the conclusions of this article will be made available by the authors on request.

## Supplementary Materials

The following supporting information can be downloaded at: https://www.mdpi.com/article/10.3390/toxics14090801/s1, Supplementary Document S1: The 827 targets of PS-NP; Supplementary Document S2: The 15,274 target of neurotoxicity; Supplementary Figure S1: GO enrichment analysis was performed on the potential targets of PS-NPs, displaying the most significantly enriched terms across three categories: Biological Process (BP), Cellular Component (CC), and Molecular Function (MF); Supplementary Figure S2: Classification of the 241 KEGG pathways into six categories: Metabolism, Genetic Information Processing, Environmental Information Processing, Cellular Processes, Organismal Systems, and Human Diseases; Supplementary Figure S3: Molecular docking of styrene with the MTOR-TFEB signaling pathway and autophagy-related and apoptosis-related target proteins; Supplementary Figure S4: The comparative analysis of day 1 vs. day 4 for scape latency, swimming distance, and swimming speed in each experimental group; Supplementary Figure S5: The analysis of nuclear/cytoplasmic or nuclear/total TFEB ratio.

## Abbreviations

The following abbreviations are used in this manuscript:

CTSB	Cathepsin B
CTSD	Cathepsin D
LAMP2	Lysosome-associated membrane protein 2
MAP1LC3B	Microtubule-associated protein 1 light chain 3 beta
MTOR	Mechanistic target of rapamycin
MWM	Morris water maze test
PNT	Position navigation test
PS-NPs	Polystyrene nanoplastics
SEM	Scanning electron microscopy
SPT	Spatial exploration test
SQSTM1	Sequestosome-1
TEM	Transmission electron microscopy
TFEB	Transcription factor EB

## Appendix A

### Appendix A.1. Materials and Methods

#### Appendix A.1.1. Characterization of PS-NPs

The morphological and surface characteristics of PS-NPs (CAS No. 9003-53-6) were characterized via scanning electron microscopy (SEM). SEM micrographs revealed the spherical geometry and surface topography of the particles [42]. Transmission electron microscopy (TEM) was employed to visualize the internal structure and ultrafine nanostructural features of PS-NPs, as well as to verify their nanoscale size. TEM images further illustrated the dispersion behavior and particle size distribution of PS-NPs. PS-NPs (80 nm) were dispersed in deionized water and subjected to ultrasonication for 10 min to achieve complete suspension before use [45]. Zeta potential measurement was performed to evaluate the surface charge of the particles and their colloidal stability in aqueous solution.

#### Appendix A.1.2. Toxicity Prediction and Analysis of PS-NPs

The keyword “polystyrene” and its monomer “styrene” were searched in the PubChem database (https://www.pubchem.ncbi.nlm.nih.gov; accessed on 3 October 2025) [46] to identify the most matching compound. Verification of the correspondence between compound names and molecular formulas was performed. The two-dimensional structure file (SDF) was downloaded, and the corresponding SMILES file was saved. Toxicity profiles were predicted using ADMETlab 3.0 (https://admetlab3.scbdd.com [47]; accessed on 3 October 2025) and ProTox-3.0 (https://tox.charite.de/protox3/index.php?site=home [48]; accessed on 3 October 2025).

#### Appendix A.1.3. Identification of PS-NP Targets

Since ChEMBL, SwissTargetPrediction and STITCH databases are primarily designed for small-molecule compounds, styrene monomer was used as the structural input of PS-NPs, given that the chemical moieties on the surface of polystyrene nanoplastics are identical to those of styrene monomers. Comparable strategies have been adopted in recent toxicological investigations of nanoplastics [25,26]. Using the obtained SMILES strings, a search was performed in the ChEMBL database with “Homo sapiens” specified as the target species to ensure that only human-relevant protein interactions were considered. To expand the pool of potential targets, we submitted the SMILES strings obtained from PubChem to the SwissTargetPrediction database (http://www.swisstargetprediction.ch/; accessed on 3 October 2025) and the STITCH database (http://stitch.embl.de/cgi/network.pl; accessed on 3 October 2025) and only retained the targets with a prediction probability greater than zero. Subsequently, the targets collected from these two databases were converted to the standardized format of targets through the UniProt database (https://www.uniprot.org/uniprotkb; accessed on 3 October 2025) and then merged and deduplicated. Furthermore, genes associated with “polystyrene” were retrieved from the CTD database and integrated with the aforementioned prediction results. All candidate targets were standardized and deduplicated using the UniProt database.

#### Appendix A.1.4. Collection and Retrieval of Neurotoxicity-Related Target Networks

Neurotoxicity-related target genes were collected from the CTD, OMIM, and GeneCards databases using the keywords “neurotoxicity syndromes”, “nerve degeneration”, “neuronal apoptosis”, and “cognitive disorder”. High-confidence target genes with a relevance score above the median were selected. The PS-NP targets and the neurotoxicity-related target genes were then combined, and after removing overlaps, the remaining genes were considered as potential targets involved in PS-NP-induced neurotoxicity.

#### Appendix A.1.5. Functional and Pathway Enrichment Analysis of Potential Targets

Using the Metascape database (https://metascape.org/gp/index.html#/main/step3; accessed on 3 October 2025) with “Homo sapiens” as the species, Gene Ontology (GO) and Kyoto Encyclopedia of Genes and Genomes (KEGG) pathway enrichment analyses were performed on potential targets of PS-NP-induced neurotoxicity. The GO terms included Cellular Components (CC), Biological Processes (BPs), and Molecular Functions (MFs). A q-value below 0.05 was considered statistically significant.

#### Appendix A.1.6. Construction of the “Toxicant–Disease–Pathway” Gene Interaction Network and Screening of Core Targets

The “toxicant–disease–pathway”-related genes were imported into the STRING database (https://string-db.org/; accessed on 3 October 2025, version 11.0) with “Homo sapiens” as the target species. A minimum interaction score threshold of “high confidence (0.700)” was set to construct the protein–protein interaction (PPI) network. The CytoHubba plugin in Cytoscape (version 3.9.1) was then used to identify core targets. Using betweenness centrality, closeness centrality, and degree (number of connections), the five most significant targets were selected [49]. Furthermore, a Venn diagram was constructed to filter the hub targets as the core targets involved in PS-NP-induced neurodegeneration.

#### Appendix A.1.7. Molecular Docking Validation

Given that conventional molecular docking software has not been developed to characterize interactions between nanoparticles and proteins, a styrene 20-mer was adopted in this study to mimic the surface chemical structure of PS-NPs for molecular docking simulations [27]. The 3D structure of styrene was obtained from PubChem. The 20-mer polystyrene structure was drawn using ChemDraw, version 22.2.0, followed by 3D-structure generation and energy minimization in Chem3D. The optimized geometric conformation was saved as a mol2 file and subsequently converted to PDBQT format with AutoDock tools. The kinase domain of *MTOR* (PDB ID: 4JSV) and full-length *TFEB* (PDB ID: 7Y62) were selected as docking receptors, as these two proteins constitute the core molecular targets of the present study. From the PDB database (https://www.rcsb.org/; accessed on 3 October 2025), the target protein structure was retrieved by setting the source organism as “Homo sapiens” and limiting the experimental method to X-ray diffraction. The PDB records that meet all of the following conditions were preferentially selected: a resolution better than 2.5 Å (1 Å = 0.1 nm), having a clear three-dimensional structure and existing in the protein–ligand complex form. The selected structures were downloaded, and they were imported into PyMOL (version 2.5.0) to remove water molecules and extract the natural ligand. Then, AutoDock Vina (version 4.4.6) was used to assign protonation states and charges, and the prepared file was subsequently stored in PDBQT format for docking. The docking results were analyzed and visualized through the PyMOL software. In this study, the styrene representative of PS-NPs shows favorable binding affinity with the core target proteins (*MTOR* and *TFEB*). More negative-binding-affinity values indicate stronger receptor–ligand interactions. A binding energy less than or equal to −5.0 kilocalories/mole (where 1 calorie is equivalent to 4.2 joules) was used as the criterion to confirm the effective interactions between the main active component and the core targe, and the results are presented visually.

#### Appendix A.1.8. Antibodies, Reagents and Chemicals

PS-NPs (80 nm) were provided by Tianjin Beisile Co., Ltd. (CAS No. 9003-53-6). RIPA lysis buffer was purchased from Beijing Solarbio Science & Technology Co., Ltd. (Beijing, China). Horseradish peroxidase (HRP)-labeled mouse anti-goat IgG and HRP-labeled rabbit anti-goat IgG were obtained from Beijing Zhongshan Jinqiao Biotechnology Co., Ltd. (Beijing, China). Antibodies against p-MTOR and MTOR were supplied by Abmart Inc. (Shanghai, China). Antibodies against TFEB, LAMP2, CTSD, CTSB, SQSTM1, MAP1LC3B, BAX, and BCL2 were purchased from Proteintech Group, Inc. (Rosemont, IL, USA).

#### Appendix A.1.9. Animal Experimentation

Specific pathogen-free (SPF) adult Sprague–Dawley (SD) rats (180–220 g, male/female ratio of 1:1) were provided by Sibefu Biotechnology Co., Ltd. (license no. SYXK (Beijing, China) 2024-0001). The animals were housed under controlled conditions (12/12 h light–dark cycle, 50–60% humidity, at 20–25 °C) in plastic cages, with free access to double-distilled water and standard pelleted chow. This work has received approval for research ethics from the Biology Ethics Committee of Shihezi University, and a proof/certificate of approval is available upon request.

Following one week of acclimation, the rats were randomly assigned to three groups (*n* = 12 per group): a control group (0 mg/kg/d), as well as low-dose (0.15 mg/kg/d) and high-dose (1.5 mg/kg/d) groups exposed to 80 nm PS-NPs. PS-NPs were administered once daily by gavage for 60 consecutive days.

The experimental doses adopted in the present study were determined based on previous research reported by Deng et al. [50]. Assuming an average human body weight of 60 kg, the daily intake of microplastics in humans is approximately 13–39.3 mg, corresponding to an intake of 0.22–0.66 mg per kilogram of body weight. The dose conversion method proposed by Nair and Jacob [51] was utilized for interspecies exposure extrapolation between humans and adult rats. For adult rats with a body weight of 0.4 kg, the Km factor was calculated as 6, while the Km value for a 60 kg adult human was 37. The equivalent dose for rats was calculated via the following formula: Animal dose (mg/kg) = Human dose (mg/kg) × (Kmhuman/Kmanimal). The calculation yielded an equivalent rat dose ranging from 1.37 to 4.07 mg/kg/day. The above conversion was performed following the body surface area (BSA)-normalized principle for pharmaceutical dose translation.

To date, there is no universally accepted standard for interspecies extrapolation of particulate pollutants such as nanoplastics. The biodistribution, tissue accumulation, and clearance kinetics of particulate matter differ fundamentally from those of soluble small-molecule pharmaceuticals. Accordingly, dose conversion solely relying on BSA normalization may fail to accurately estimate equivalent exposure to doses of nanoplastics across distinct species.

Given the uncertainties associated with BSA-based extrapolation, together with the commonly used dose ranges documented in existing toxicological investigations of PS-NPs, two low-exposure doses (0.15 mg/kg and 1.5 mg/kg) were finally selected in this work. A 60-day chronic low-dose-exposure protocol was designed herein, primarily because NPs tend to accumulate in organisms. Chronic low-level exposure is considered more environmentally relevant compared with acute high-dose exposure. Furthermore, detectable functional impairments related to neurodegenerative alterations (e.g., impaired learning and memory capacity) generally require prolonged exposure durations to develop, and this experimental design could better recapitulate the real-world exposure dosage and duration in human populations.

Learning and memory were assessed using the Morris water maze (MWM) test (six rats per group, half male and half female, were randomly selected for the MWM test). After the MWM test, these rats were euthanized, and the hippocampal tissues were collected on ice. The remaining samples were wrapped in aluminum foil, flash-frozen in liquid nitrogen for 15 s, and stored at −80 °C for further analyses.

#### Appendix A.1.10. MWM Test

The MWM setup consisted of a black circular pool (50 cm in height and 180 cm in diameter) filled with water to a depth of approximately 32 cm and maintained at 22 ± 1 °C. A cylindrical platform (20 cm in height and 8 cm in diameter) was positioned in the center of the third quadrant and kept in a fixed location throughout the experiment. A video recording system was mounted above the pool, and the pool was divided into four equal quadrants. The test included a place navigation test (PNT) and a spatial probe test (SPT).

##### PNT

The PNT was conducted over the first four days. Each rat was introduced into the water facing the pool wall, sequentially from the entry points of quadrants I, II, III, and IV in a clockwise order. The escape latency, defined as the time required to locate the hidden platform within 60 s, was measured. If the rat reached the platform within 60 s, the actual latency was recorded; if not, the experimenter guided it to the platform, where it remained for 10 s, and a maximum score of 60 s was assigned. Escape latency, swim distance, swim speed, and swim path were recorded to assess spatial learning and memory.

##### SPT

On the day after the PNT, the SPT was carried out. The platform was removed, and the rat was released into the water facing the wall at the same starting point as in quadrant I. Within 60 s, the number of crossings over the former platform location, the dwell time and distance traveled in the target quadrant, and the swimming trajectory were documented to evaluate memory storage and retrieval.

#### Appendix A.1.11. Western Blot Analysis

Western blotting was employed to assess the expression levels of p-MTOR, MTOR, total TFEB, nuclear TFEB, cytoplasmic TFEB, lysosomal function-related proteins (LAMP2, CTSD, and CTSB), autophagy-related proteins (MAP1LC3B and SQSTM1), and apoptosis-related proteins (BAX and BCL2) in the rat hippocampus. Hippocampal tissues were homogenized in RIPA buffer supplemented with 1% protease inhibitor, and the total protein concentration was quantified. Equal amounts of protein were separated by sodium dodecyl sulphate–polyacrylamide gel electrophoresis (SDS-PAGE) and subsequently transferred to polyvinylidene fluoride (PVDF) membranes using an electrophoresis transfer apparatus. These PVDF membranes were blocked at room temperature for 2 h in Tris buffer containing 5% skimmed milk powder and then incubated overnight (14–16 h) at 4 °C with primary antibodies against the following: β-actin, 1:5000; GAPDH, 1:5000; p-MTOR, 1:1000; MTOR, 1:1000; TFEB, 1:1000; LAMP2, 1:1000; CTSD, 1:1000; CTSB, 1:1000; SQSTM1, 1:1000; MAP1LC3B, 1:1000; BAX, 1:1000; and BCL2, 1:1000. Following the washing step, the membranes were incubated with the corresponding secondary antibodies (1:20,000) for 2 h at room temperature. Protein bands were visualized by means of enhanced chemiluminescence (ECL) reagents and a chemiluminescence imaging system. Quantification of band intensities was carried out using the ImageJ software (version 1.7.0), and graphs were generated with GraphPad Prism (version 8.0.2.263).

#### Appendix A.1.12. Statistical Analysis

Statistical analyses were performed using the SPSS software (version 26.0). MWM data were evaluated by repeated-measures analysis of variance (ANOVA). For the remaining datasets, multiple comparisons were conducted using one-way ANOVA with the Student–Newman–Keuls (SNK) post hoc test. Statistical significance was set at *p* < 0.05, and all data are presented as the mean ± standard deviation (SD).
