# Supplementary material for: Network Toxicology Integrates In Vivo Evidence Linking Impaired Autophagic Clearance Involving the MTOR-TFEB Axis to Polystyrene Nanoplastic-Induced Neurotoxicity
Source: Toxics. 2026 Sep 9;14(9):801. doi: 10.3390/toxics14090801 (PMC13611230; doi:10.3390/toxics14090801)
Supplement: Supplementary file 1 [file toxics-14-00801-s001.zip › Figure legend.pdf]

Supplementary Figure S1. GO enrichment analysis was performed on the potential targets of PS-NPs, displaying the most significantly enriched terms across three categories: Biological Process (BP), Cellular Component (CC), and Molecular Function (MF). The horizontal axis represents $-\log_{10}$(p-value), with higher values indicating greater enrichment significance ($p < 0.05$ was considered statistically significant). The results showed that BP terms were mainly enriched in mitochondrial ATP synthesis coupled electron transport, aerobic electron transport chain, and oxidative phosphorylation; CC terms were enriched in NADH dehydrogenase complex, respiratory chain complex I, and mitochondrial membrane; MF terms were enriched in NADH dehydrogenase activity, oxidoreduction-driven active transmembrane transporter activity, and protein kinase activity. The analysis was performed using the Metascape platform, with q-value < 0.05 as the threshold for significant enrichment.

Supplementary Figure S2. Classification of the 241 KEGG pathways into six categories: Metabolism, Genetic Information Processing, Environmental Information Processing, Cellular Processes, Organismal Systems, and Human Diseases.

Supplementary Figure S3. Molecular docking of styrene with the MTOR-TFEB signaling pathway and autophagy-related and apoptosis-related target proteins. (A) Molecular docking models of styrene with MTOR . (B) Molecular docking models of styrene with TFEB.(C) Molecular docking models of styrene with LAMP2. (D) Molecular docking models of styrene with CTSD. (E) Molecular docking models of styrene with CTSB. (F) Molecular docking models of styrene with SQSTM1.(G) Molecular docking models of styrene with MAP1LC3B. (H) Molecular docking models of styrene with BCL2. (I) Molecular docking models of styrene with BAX. (J) Core target proteins: PDB IDs and docking parameters.

Supplementary Figure S4. The comparative analysis of day 1 vs day 4 for scape latency, swimming distance, and swimming speed in each experimental group. (A) The

comparative analysis of day 1 vs day 4 for scape latency. (B) The comparative analysis of day 1 vs day 4 for swimming distance. (C) The comparative analysis of day 1 vs day 4 for swimming speed.

Supplementary Figure S5. The analysis of nuclear/cytoplasmic or nuclear/total TFEB ratio. (A) The analysis of nuclear/cytoplasmic TFEB ratio. (B) The analysis of nuclear/total TFEB ratio.
